# Intracorporeal versus extracorporeal anastomosis in laparoscopic right colectomy: a retrospective study

**DOI:** 10.1186/s12957-023-03023-8

**Published:** 2023-05-20

**Authors:** Fangqian Chen, Zeping Lv, Wenqing Feng, Zhuoqing Xu, Yiming Miao, Zifeng Xu, Yuchen Zhang, Han Gao, Minhua Zheng, Yaping Zong, Jingkun Zhao, Aiguo Lu

**Affiliations:** 1 Department of General Surgery, Ruijin Hospital, Shanghai Jiao Tong University School of Medicine, Shanghai, 200025 China; 2 Shanghai Minimally Invasive Surgery Center, Ruijin Hospital, Shanghai Jiao Tong University School of Medicine, Shanghai, 200025 China

**Keywords:** Anastomosis, Intracorporeal, Extracorporeal, Laparoscopic right colectomy, Totally laparoscopic right colectomy, Laparoscopy-assisted right colectomy

## Abstract

**Background:**

The surgical procedure for laparoscopic right colectomy (LRC) is not standardized. Some published studies show the superiority of ileocolic anastomosis (IIA), but the evidence so far is insufficient. This study aimed to investigate the potential advantages in postoperative recovery and safety of IIA in LRC.

**Methods:**

A total of 114 patients who underwent LRC with IIA (*n* = 58) or extracorporeal ileocolic anastomosis (EIA, *n* = 56) between January 2019 and September 2021 were enrolled. We collected certain factors as clinical features, intraoperative characteristics, oncological outcomes, postoperative recovery, and short-term outcomes. Our primary outcome was time to gastrointestinal (GI) function recovery. Secondary outcomes were postoperative complications within 30 days, postoperative pain, and length of hospital stay.

**Results:**

Faster GI recovery and less postoperative pain were observed in patients with IIA compared to EIA [time to first flatus: (2.4 ± 0.7) vs (2.8 ± 1.0) days, *p* < 0.01; time to liquid intake: (3.5 ± 0.7) vs (4.0 ± 1.1) days, *p* = 0.01; postoperative visual analogue scale score: (3.9 ± 1.0) vs (4.3 ± 0.6), *p* = 0.02]. No significant differences were detected in oncological outcomes or postoperative complications. IIA, rather than EIA, tended to be performed in patients with higher body mass index [(23.93 ± 3.52) vs (22.36 ± 2.87) kg/m^2^, *p* = 0.01].

**Conclusions:**

IIA is associated with faster GI function recovery and less postoperative pain and may be more favorable for obese patients.

## Introduction

Colectomy is the primary treatment for nonmetastatic colon cancer (CC) [1]. Since Jacobs first reported laparoscopically assisted colectomy (LAC) in 1991, surgical techniques for LAC have developed rapidly around the world. LAC has statistically and clinically significant advantages over open surgery in respect of enhancing the recovery of gastrointestinal (GI) function, reducing intraoperative blood loss, postoperative pain, and length of hospital stay (LOHS), while maintaining similar overall survival [2–4]. Laparoscopic colectomy has become a standardized treatment for CC, adopted by surgeons worldwide [5–7]. However, the surgical procedure for laparoscopic right colectomy (LRC) is not standardized. LRC can be divided into laparoscopy-assisted right colectomy (LARC) and total laparoscopic right colectomy (TLRC), according to the method of reconstruction of the digestive tract continuity. The initial steps are similar in the two surgical methods, whereas extracorporeal ileocolic anastomosis (EIA) and intracorporeal ileocolic anastomosis (IIA) are performed in LARC and TLRC, respectively. TLRC requires more expertise and technical support, and some studies reported that it costs more operative time than LARC [8, 9]. Some published studies show the superiority of IIA, while others show the opposite [10–13]. The evidence so far was insufficient. The aim of this study was to investigate the potential advantages and safety of IIA in LRC.

## Methods

### Patients

A total of 114 consecutive patients with right-sided colon neoplasms and underwent LRC in the minimally invasive surgery center of Ruijin Hospital affiliated to Shanghai Jiao Tong University, School of Medicine, Shanghai, China, from January 2019 and September 2021 were included.

The exclusion criteria were as follows: (1) age ≤ 18 or > 75 years old, (2) distant metastases, (3) emergency surgery for acute abdominal complications (including acute bowel obstruction and perforation), (4) ASA score > 3, (5) preoperative chemotherapy or chemoradiotherapy, and (6) synchronous resection of separate intestinal segments. All LRCs were primarily performed by one skilled surgeon, who was fully trained in laparoscopic colorectal surgery with an annual volume of over 200 procedures. The surgeon was trained to perform IIA in LRC 6 months prior to the study period and had overcome learning curve effects.

### Surgical technique

Patients underwent TLRC or LARC depending on the surgeon’s previous individual experience and clinical considerations.

The preparation for surgery, patient position, surgeon location, and insertion of trocars were the same as previously reported [14]. For patients with malignant tumors, the mesentery and vessels were dissected and separated following the principle of complete mesocolic excision in the two groups.

**Fig. 1:**
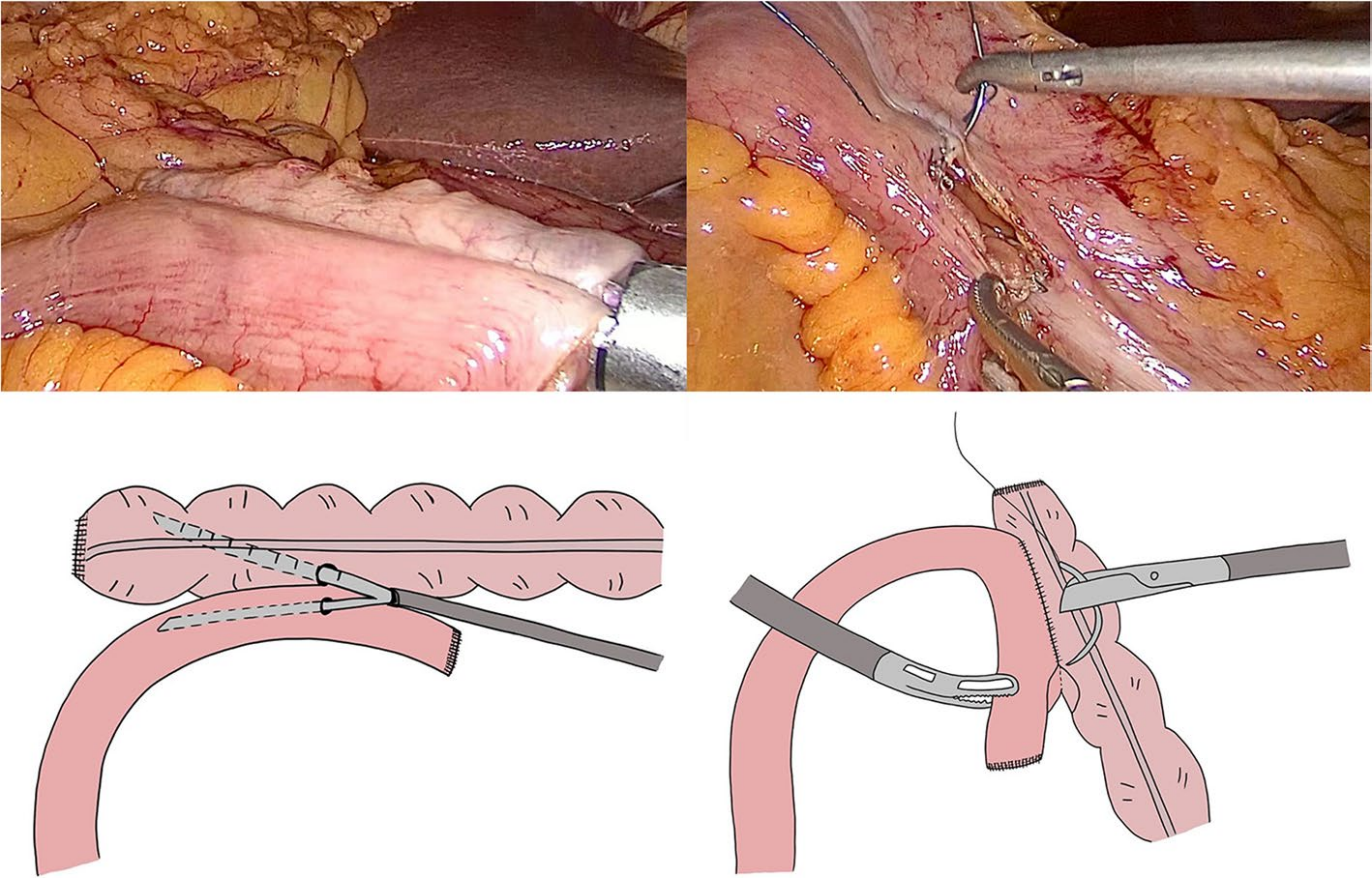
Key steps for IIA. **A** Isoperistaltic anastomosis using a linear stapler. **B** Enterotomy closure using a barbed suture. **C** Schematic diagram of isoperistaltic anastomosis. **D** schematic diagram of enterotomy closure in TLRC

**Fig. 2:**
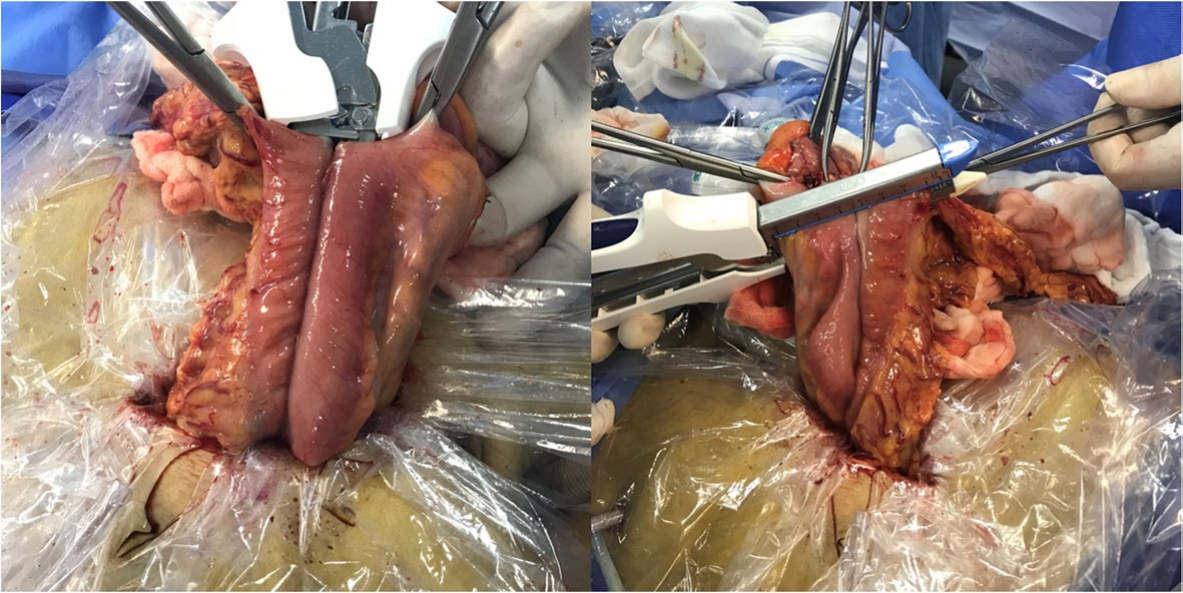
Key steps for EIA. **A** Side-to-side anastomosis using a linear cutter. **B** Enterotomy closure using a linear cutter

The method of transection and anastomosis varied according to the procedure selected (TLRC or LARC). Key steps for IIA and EIA are as follows:

- IIA group (Fig. 1): (1) Dissociation of the mesentery in the abdominal cavity, (2) transection of terminal ileum and transverse colon using a linear stapler, (3) isoperistaltic anastomosis using a linear stapler, and (4) enterotomy closure with double-layer sutures: a running barbed suture was used for the first layer, and a 3–0 Vicryl interrupted suture was used for the second layer.
- EIA group (Fig. 2): (1) Dissociation of the mesentery in the abdominal cavity, (2) bowel extraction through an enlargement of the skin incision in the paraumbilical position and transection of terminal ileum and transverse colon, (3) side-to-side anastomosis using a linear cutter, (4) enterotomy closure using the linear cutter, and (5) anastomosis reinforcement using 3–0 Vicryl interrupted suture.

### Data collection

We retrospectively collected data about clinical features, intraoperative characteristics, oncological outcomes, postoperative recovery, and short-term outcomes in the electronic medical record system. Intraoperative characteristics included operative time and intraoperative blood loss. Oncological outcomes included number of lymph nodes harvested and margin distance (proximal and distal). Postoperative recovery included GI function recovery (time to first flatus, time to first defecation, time to liquid intake), postoperative pain, and LOHS. Postoperative pain was assessed from the day of surgery to the day of discharge. All nurses in our institution have been trained to evaluate postoperative pain by visual analogue scale (VAS) (0–10, handheld slide rule type) [15]. Patients who underwent bowel surgery in our institution are asked to promptly inform nurses of flatus and defecation. During daily ward rounds, the surgeon would instruct patients about diet (fasting, drinking, liquid diet or semisolid diet) based on the recovery of GI function and tolerance of current diet (no nausea or vomiting over 24 h). Short-term outcomes referred to complications within 30 days after surgery.

Our primary outcome was time to recovery of GI function measured as time to flatus. Secondary outcomes were time to first defecation, time to liquid intake, postoperative complications within 30 days, postoperative pain, and LOHS.

### Statistical analysis

Categorical data are presented as absolute numbers and percentages, whereas continuous variables are presented as means ± standard deviations (SDs). The Pearson’s chi-squared test was used for the comparison of categorical variables and Student’s *t*-test for the analysis of continuous variables. A multivariate linear regression (MLR) was performed to assess the impact of the surgical procedure and other factors in the baseline data on the primary outcome. A two-sided significance level less than 0.05 was considered to indicate statistical significance. All statistical analyses were performed using the Statistical Package for Social Sciences (SPSS) version 26 (IBM Corp., Armonk, New York, USA) and R version 4.2.0 (R Foundation for Statistical Computing, Vienna, Austria).

## Results

A total of 114 consecutive patients were included in the study with 58 patients in the IIA group and 56 patients in the EIA group, respectively. The IIA group involved 26 males and 32 females with a mean age of 61 years old. Respectively, 14, 31, and 13 patients were diagnosed with tumors of the cecum, ascending colon, and hepatic flexure. The IIA group included 8 benign cases and 50 malignant cases. As for the EIA group, 30 males and 26 females with a mean age of 62 years old were involved. Respectively, 13, 32, and 11 patients were diagnosed with tumors located in cecum, ascending colon, and hepatic flexure. The group consisted of 10 benign cases and 46 malignant cases. Demographic features were displayed in Table 1. No significant differences were detected in terms of sex, age, hemoglobin level, albumin level, tumor type, tumor site, or tumor staging between the IIA and EIA groups. Higher BMI was observed in the IIA group.

**Table 1:** Demographic features of the study cohort

	IIA (*n* = 58)	EIA (*n* = 56)	*p*-value
**Sex**			0.35
Male	26 (44.8%)	30 (53.6%)	
Female	32 (55.2%)	26 (46.4%)	
**Age (yrs)**	61 ± 11	62 ± 11	0.48
**BMI (kg/m**^**2**^**)**	23.93 ± 3.52	22.36 ± 2.87	0.01
**Hb (g/L)**	122.1 ± 23.8	117.1 ± 22.7	0.26
**Ab (g/L)**	40.2 ± 4.3	38.9 ± 4.8	0.12
**Tumor type**			0.94
Benign	8 (13.8%)	10 (17.9%)	
Malignant	50 (86.2%)	46 (82.1%)	
**Tumor site**			0.91
Cecum	14 (24.1%)	13 (23.2%)	
Ascending colon	31 (53.4%)	32 (57.1%)	
Hepatic flexure	13 (22.5%)	11 (19.7%)	
**Tumor staging**			0.06
I	17 (34.0%)	6 (13.0%)	
II	19 (38.0%)	24 (52.2%)	
III	14 (28.0%)	16 (34.8%)	

*Hb* Hemoglobin, *Ab* albumin

Intraoperative characteristics were shown in Table 2. The mean operative time was 130 ± 32 min in the IIA group and 125 ± 29 min in the EIA group. Thirty out of 114 patients experienced over 100-ml intraoperative blood loss with 12 who underwent IIA and 18 underwent EIA. No intraoperative complications, such as ureter injury, bowel injury, and subcutaneous emphysema, were observed in any patients.

**Table 2:** Intraoperative characteristics

	IIA (*n* = 58)	EIA (*n* = 56)	*p*-value
**Operative time(min)**	130 ± 32	125 ± 29	0.34
**Intraoperative blood loss (mL)**			0.24
≥ 100	12	18	
< 100	46	38	

Oncologic outcomes of malignant cases were analyzed. In the number of lymph nodes harvested, the mean proximal margin distance and the mean distal margin distance were comparable in the IIA and EIA groups (Table 3), indicating good specimen quality.

**Table 3:** Oncological outcomes of malignant cases

	IIA (*n* = 50)	EIA (*n* = 46)	*p*-value
**Number of lymph nodes harvested**	21 ± 10	21 ± 6	0.97
**Margin distance (cm)**			
Proximal	12.2 ± 7.9	10.4 ± 5.1	0.18
Distal	10.3 ± 5.2	10.5 ± 5.4	0.87

**Fig. 3:**
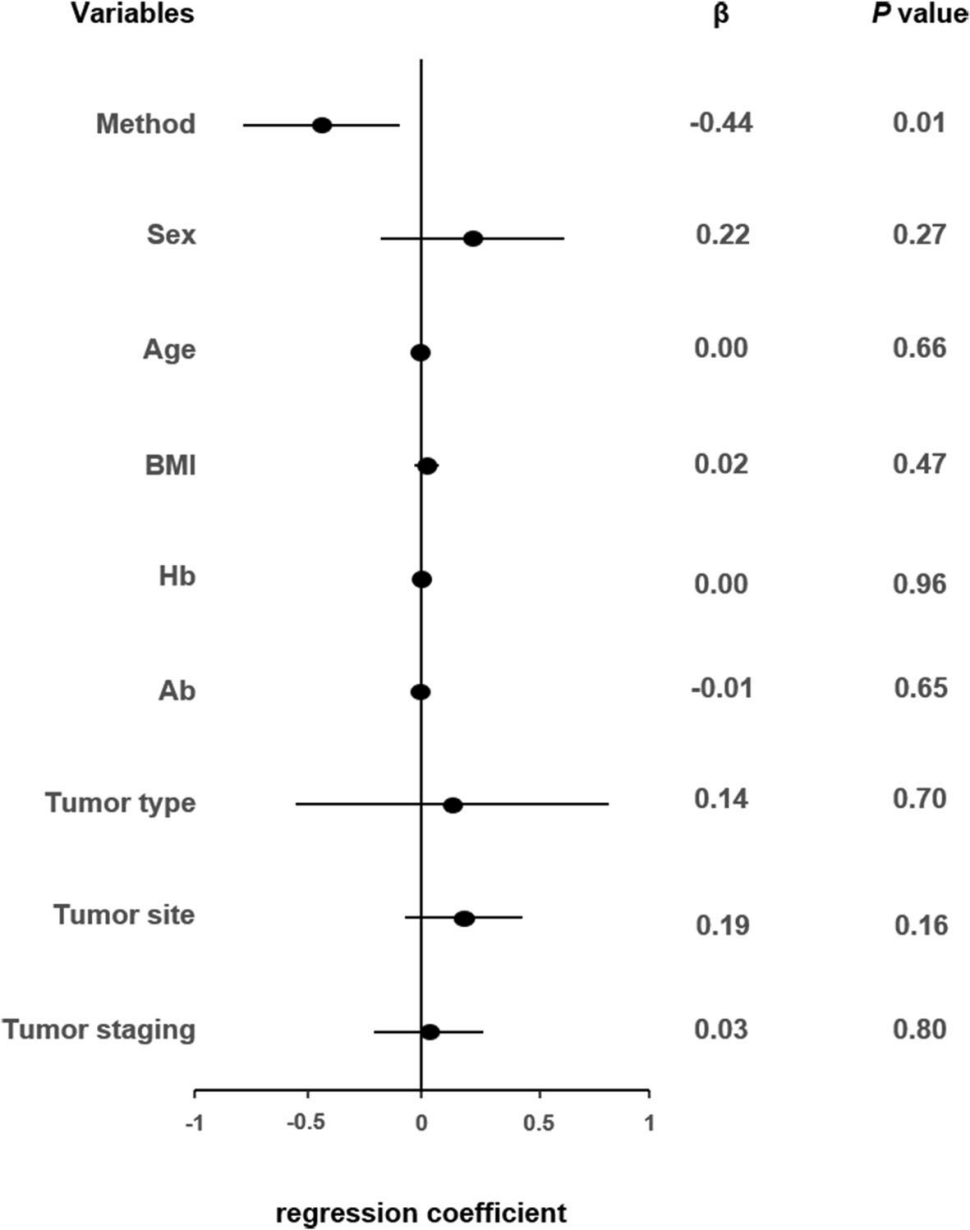
Forest plot displaying time to first flatus of multivariate linear regression for method, sex, age, BMI, Hb, Ab, tumor type, tumor site, and tumor staging

A MLR was performed to assess the impact of the surgical approach and other factors in the baseline data on the primary outcome (Fig. 3). The MLR showed that the method of reconstruction of the digestive tract continuity (IIA or EIA) was the only independent risk factor for time to first flatus (*p* = 0.01). Table 4 demonstrates postoperative outcomes of the two groups. Patients in the IIA group showed a quicker recovery of GI function and less postoperative pain than the EIA group. The mean time to first flatus in patients undergoing IIA was 2.4 ± 0.7 days, and the mean time to liquid intake was 3.5 ± 0.8 days. Lower VAS score was detected in the IIA group, especially on postoperative day (POD) 0 (3.9 ± 1.0, *p* = 0.02) and POD 2 (2.4 ± 0.6 *p* = 0.02). No significant difference was found in LOHS between the two groups. Postoperative complications were graded according to the Clavien-Dindo classification. There was a case of anastomotic bleeding recorded in the EIA group. The patient received blood transfusion without invasive intervention and was successfully discharged on POD 12. There was no case of anastomotic leak (the incidence of anastomosis-related complications was < 2% at our institution [16]). Other postoperative complications were recorded in detail. There was one case of paralytic bowel obstruction, one case of pneumonia, and one case of wound infection which were readmitted within 30 days in the IIA group. One case of chyme leak and one case of delayed recovery of GI function are in the EIA group. All five cases of complications were Clavien-Dindo grades 1–2. No grades 3–4 complications occurred in our study. There was no significant difference in complications within 30 days after surgery between the two groups. A subgroup analysis of patients with *BMI* ≥ 24 kg/m^2^ was performed (Table 5), showing no significant difference in clinical characteristics and complications within 30 days after surgery between the two groups.

**Table 4:** Postoperative results

	IIA (*n* = 58)	EIA (*n* = 56)	*p*-value
**Return of GI function (days)**			
Time to first flatus	2.4 ± 0.7	2.8 ± 1	< 0.01
Time to first defecation	3.8 ± 1.5	4.2 ± 1.8	0.22
**Time to liquid intake (days)**	3.5 ± 0.8	4.0 ± 1.1	0.01
**VAS score**			
POD 0	3.9 ± 1.0	4.3 ± 0.6	0.02
POD 1	3.2 ± 1.1	3.5 ± 0.7	0.08
POD 2	2.4 ± 0.6	2.7 ± 0.7	0.02
POD 3	2.3 ± 0.4	2.4 ± 0.7	0.23
POD 4	2.2 ± 0.4	2.1 ± 0.5	0.53
POD 5	2.0 ± 0.4	1.9 ± 0.6	0.83
**LOHS (days)**	6.9 ± 2.9	7.0 ± 1.6	0.84
**Anastomosis-related complication**			0.31
Anastomotic bleeding	0 (0%)	1 (1.8%)	
Anastomotic leak	-	-	
**Type of other complication**			0.68
Bowel obstruction	1 (1.7%)	-	
Delayed recovery of GI function	-	1 (1.8%)	
Chyme leak	-	1 (1.8%)	
Pneumonia	1 (1.7%)	-	
Wound infection	1 (1.7%)	-	

*POD* Postoperative day, POD 0 means the day for surgery

**Table 5:** Subgroup analysis of patients with *BMI* ≥ 24 kg/m^2^

	IIA (*n* = 29)	EIA (*n* = 15)	*p*-value
**Gender**			0.07
Male	15 (51.7%)	12 (80.0%)	
Female	14 (48.3%)	3 (20.0%)	
**Age (yrs)**	61 ± 10	60 ± 10	0.74
**BMI (kg/m**^**2**^**)**	26.61 ± 2.48	25.96 ± 1.85	0.38
**Hb (g/L)**	124.1 ± 26.7	128.7 ± 20.4	0.56
**Ab (g/L)**	40.1 ± 3.9	39.9 ± 4.3	0.88
**Tumor type**			0.46
Benign	5 (17.2%)	4 (26.7%)	
Malignant	24 (82.8%)	11 (73.3%)	
**Tumor site**			0.73
Cecum	7 (24.1%)	4 (26.7%)	
Ascending colon	13 (44.8%)	8 (53.3%)	
Hepatic flexure	9 (31.1%)	3 (20.0%)	
**Tumor staging**			0.19
I	6 (25.0%)	0 (0.0%)	
II	11 (45.8%)	7 (63.6%)	
III	7 (29.2%)	4 (36.4%)	
**Type of other complication**			0.30
Bowel obstruction	1 (3.4%)	-	
Wound infection	1 (3.4%)	-	

## Discussion

For surgical procedures in LRC, IIA or EIA is debatable. Certain studies [10, 11, 17] indicated that both surgical procedures had similar pathological outcomes and long-term outcomes, including overall survival, disease-free survival, and the rate of peritoneal recurrence. IIA was even found to achieve a more precise tumor excision than EIA [17]. Some studies [12, 17–20] and meta-analyses [21, 22] concluded that patients who underwent IIA experienced a faster recovery of GI function, less postoperative pain, lower surgical stress response (SSR), fewer medical complications, and shorter LOHS. In contrast, some studies did not support the superiority of IIA over EIA [23, 24]. Our study now provides evidence for the advantages in postoperative recovery and safety of IIA.

Thirty out of 114 patients experienced over 100-ml intraoperative blood loss, with 12 who underwent IIA and 18 underwent EIA. Although the difference (*p* = 0.24) did not reach significance owing to the small size of our study cohort, in performing EIA, we noticed potential risk of bleeding from excessive traction of mesentery due to inadequate bowel freeing. This was particularly evident in obese patients with thicker subcutaneous abdominal fat and relatively shorter mesentery, where bleeding due to excessive traction of mesentery tended to be more insidious during EIA. Therefore, the surgeon in our retrospective study tended to perform IIA in patients with higher BMI. It was hypothesized that IIA might decrease the incision length, reduce conversion rate, and eliminate the need for bowel exteriorization for anastomosis, so it might be particularly beneficial for obese patients [25, 26]. Some published studies [27–30] show that obesity is associated with postoperative complications, anastomotic leakage, and reoperation. A negative influence of visceral fat on lymph nodes harvested was observed in patients with colorectal cancer [28]. In contrast, a case-matched study [31] concluded that IIA in patients with obesity (*BMI* > 30 kg/m^2^) was associated with similar short-term outcomes and lower incidence of incisional hernias compared to EIA and might reduce the risk of hospital readmission. In our subgroup analysis of patients with *BMI* ≥ 24 kg/m^2^, no significant difference was found in rate or severity of postoperative complications between the two groups. Besides, the oncological outcomes in the IIA group were similar to those in the EIA group. Considering the potential advantages of reduced intraoperative risk, we believe that IIA could be a better approach in obese patients.

Under the wide and clear view in TLRC, anastomosis twists are more likely to be avoided. Furthermore, with the evolution of advanced laparoscopic linear staplers, IIA procedure has become simpler and more efficient. Although IIA poses greater technical difficulty and requires advanced technical skills in laparoscopic surgery, we believe that with some training, surgeons can complete TLRC successfully without increasing operative time. In our study, the time to first flatus was deemed as a marker of GI function recovery in patients who underwent surgery. Time to first flatus was significantly shorter in the IIA group compared to the EIA group (*p* < 0.01). The mean time of first defecation was shorter in the IIA group, but did not reach statistical significance (*p* = 0.22). One hypothesis was that all patients emptied their bowel contents preoperatively and ate relatively little postoperatively; the formation of stool was interfered in both groups. Meanwhile, time to liquid intake occurred earlier in patients undergoing IIA (*p* < 0.01). Thus, we inferred that owing to less exteriorization and dissection of bowel and mesentery in IIA, TLRC had a smaller effect on GI motility.

In our study, the surgeon adopted a double-layer enterotomy closure technique, in which a running barbed suture was used for the first layer, and a 3–0 Vicryl interrupted suture was used for the second layer, to fashion an IIA. Previous studies have shown that the use of barbed sutures for enterotomy closure is safe and efficient, and a double-layer closure technique can significantly reduce the incidence of anastomotic leakage compared to the single-layer closure technique [32, 33]. Milone et al. have demonstrated that the use of a running barbed suture in the first layer can significantly reduce bleeding and leakage, while the type of suture thread (braided, non-braided, and barbed) and the method of suturing (running or interrupted) in the second layer did not have a significant effect on bleeding and leakage [34]. Our results showed no anastomotic bleeding or leakage in the IIA group, indicating that our approach to performing IIA was safe and effective.

Patients who underwent IIA suffered less postoperative pain, particularly on the POD 0 (*p* = 0.02) and POD 2 (*p* = 0.02). The mean VAS score on POD 1 tended to be lower in the IIA group, without statistical significance. As shown in Table 4, patients experienced significantly less pain from POD 3 onward, and there was no difference in VAS scores between the two groups. The benefit of IIA over EIA in reducing postoperative pain especially within 48 h may be associated with a shorter skin incision for specimen extraction. Data on the length of skin incision was not recorded in our hospital’s electronic medical record system, but this has been confirmed in some other studies [12].

In our study, there was no significant difference in LOHS between the two groups, despite less postoperative pain and faster recovery of GI function in the IIA group. Since our team were very cautious about postoperative complications, especially anastomotic leaks, which often occurred 5–7 days after surgery, we preferred to discharging them after making sure that an anastomotic leak was unlikely to occur. Significant difference in LOHS may be reached within an enhanced recovery (ERAS) program with a different discharge principle.

This study has some limitations. First, the study was limited by its retrospective, single-institution, and single-surgeon nature. Second, the data on postoperative complications included only those during hospitalization, but not the mid- and long-term follow-up outcomes, such as incisional hernia, survival, and recurrence after discharge. Lastly, the low incidence of postoperative complications may suggest that this study is underpowered to identify statistical differences. In order to mitigate these drawbacks, we have designed an RCT which is currently in enrollment. The study was registered with the Chinese Clinical Trials Registry (ChiCTR2100053282). All patients were provided written informed consent before enrollment. The study protocol was approved by the Ruijin Hospital Ethics Committee (Shanghai Jiao Tong University School of Medicine).

## Conclusions

LRC with IIA may be associated with faster GI function recovery and less postoperative pain, with comparable oncological outcomes compared to EIA. IIA may be more favorable for obese patients.

## Data Availability

The data that support the findings of this study are available from the Department of General Surgery, Ruijin Hospital, Shanghai Jiao Tong University School of Medicine, but restrictions apply to the availability of these data, which were used under license for the current study, and so are not publicly available. Data are however available from the authors upon reasonable request and with permission of Department of General Surgery, Ruijin Hospital, Shanghai Jiao Tong University School of Medicine.

## Abbreviations

BMI: Body mass index
CC: Colon cancer
EIA: Extracorporeal ileocolic anastomosis
ERAS: Enhanced recovery after surgery
GI: Gastrointestinal
IIA: Intracorporeal ileocolic anastomosis
LAC: Laparoscopically assisted colectomy
LARC: Laparoscopic-assisted right colectomy
LOHS: Length of hospital stay
LRC: Laparoscopic right colectomy
POD: Postoperative day
SD: Standard deviation
SSR: Surgical stress response
TLRC: Total laparoscopic right colectomy
VAS: Visual analogue scale score
